# Persistent Polyclonal B Cell Lymphocytosis B Cells Can Be Activated through CD40-CD154 Interaction

**DOI:** 10.1155/2014/854124

**Published:** 2014-12-14

**Authors:** Emmanuelle Dugas-Bourdages, Sonia Néron, Annie Roy, André Darveau, Robert Delage

**Affiliations:** ^1^Centre Universitaire d'Hématologie et d'Oncologie de Québec, CHU de Québec, Hôpital de l'Enfant-Jésus, 1401 18ième rue, Québec, QC, Canada G1J 1Z4; ^2^Héma-Québec, Recherche et Développement, 1070 avenue des Sciences-de-la-Vie, Québec, QC, Canada G1V 5C3; ^3^Département de Biochimie, de Microbiologie et de Bio-Informatique, Pavillon Alexandre-Vachon, 1045 avenue de la Médecine, Bureau 3428, Université Laval, Québec, QC, Canada G1V 0A6

## Abstract

Persistent polyclonal B cell lymphocytosis (PPBL) is a rare disorder, diagnosed primarily in adult female smokers and characterized by an expansion of CD19^+^CD27^+^IgM^+^ memory B cells, by the presence of binucleated lymphocytes, and by a moderate elevation of serum IgM. The clinical course is usually benign, but it is not known whether or not PPBL might be part of a process leading to the emergence of a malignant proliferative disorder. In this study we sought to investigate the functional response of B cells from patients with PPBL by use of an optimal memory B cell culture model based on the CD40-CD154 interaction. We found that the proliferation of PPBL B cells was almost as important as that of B cells from normal controls, resulting in high immunoglobulin secretion with *in vitro* isotypic switching. We conclude that the CD40-CD154 activation pathway is functional in the memory B cell population of PPBL patients, suggesting that the disorder may be due to either a dysfunction of other cells in the microenvironment or a possible defect in another B cell activation pathway.

## 1. Introduction

Persistent polyclonal B cell lymphocytosis (PPBL) is a rare and presumably nonmalignant lymphoproliferative disorder diagnosed predominantly in women [1, 2], although a few men have also been diagnosed with this condition [3–5]. Clinical symptoms are nonspecific except for mild fatigue in most individuals with this disorder [1, 6]. Patients, usually cigarette smokers, present with elevated polyclonal serum IgM and a persistent polyclonal lymphocytosis of memory B cell origin as evidenced, on flow cytometry, by a population of CD27^+^IgM^+^IgD^+^ cells with normal *κ*/*λ* ratio [7–11] representing more than 70% of their total B lymphocytes [12]. The blood smear in these patients is characterized by the presence of mostly atypical lymphocytes with abundant cytoplasm and mature nuclei. Binuclearity can be observed in 1–9% of their lymphocytes [13]. Patients predominantly express the HLA-DR7 phenotype, while this particular allele usually occurs in only 26% of the normal Caucasian population [14].

The clinical course is usually benign, but we have previously described the case of one individual who developed a diffuse large-B-cell lymphoma (DLBCL) 19 years after a diagnosis of PPBL [15]. Overall, a small proportion of patients with PPBL has been reported in the literature to have developed a malignant disease [16–18]. Although the pathophysiology of this disorder remains largely unknown, a familial link is one of its constant features, suggesting the existence of an underlying genetic defect [19]. Despite the apparent polyclonal nature of the B cell proliferation, the frequency of rearrangements between the* bcl-2* and Ig heavy chain genes is 100-fold greater than that observed in normal B cells, and multiple* bcl-2/Ig* gene rearrangements have been observed in all PPBL patients [20]. An isochromosome 3q+ (i3)(q10) has also been described in a varying proportion of the B cell population [3, 18]. Such genetic aberrations were always restricted to the B cells, indicating the presence of a distinct clonal cytogenetic population in PPBL patients [3]. This confirms that some B cells in this disorder are distinct from their normal counterparts. However, sparse information is as yet available on the functional properties of B cells in PPBL.

It has been shown that PPBL B cells are memory cells presenting the CD27^+^IgM^+^IgD^+^ immunotype [11, 21] with a large repertoire diversity [11, 22] and that they could originate from the B cell populations of the splenic marginal zone [23]. Marginal zone CD27^+^IgM^+^IgD^+^ B cells likely are memory cells that can be generated independently from a germinal center reaction and T cell help, while also being able to respond to the CD40-CD154 interaction [24, 25]. The binding of CD40 to CD154 expressed on activated T cells plays a central role in B cell activation, proliferation, and immunoglobulin isotype switching [26]. B lymphocytes from healthy controls grow perfectly well in a culture system based on this interaction in the presence of IL-4 [26, 27]. However, we have previously shown that PPBL B lymphocytes were unable to proliferate following* in vitro* CD40-CD154 interaction. These observations were suggestive of a possible defect in the CD40 pathway, although CD40 expression, sequencing, and tyrosine phosphorylation appeared to be normal [28]. Others have reported later that the circulating CD19^+^CD27^+^ memory B cells from normal individuals were unresponsive to high-level CD40-CD154 interaction [29]. Finally, it has been shown that a reduced-intensity CD40-CD154 interaction in the presence of IL-2, IL-4, and IL-10 results in the proliferation, expansion, and immunoglobulin secretion of normal memory CD19^+^CD27^+^ B cells [30, 31].

Since PPBL B cells share the CD27 expression of normal memory B cells, we have designed a study to investigate the response of B lymphocytes from patients with PPBL in cultures with low-intensity CD40-CD154 interaction and to further characterize these cells, especially their isotype switching and immunoglobulin secretion.

## 2. Patients and Methods

### 2.1. Patients and Healthy Controls

This study was conducted on six female patients (8010, 8011, 8013, 8030, 8031, and 8032) ranging from 47 to 69 years. All individuals were asked to answer a questionnaire inquiring about their habits and health status. PPBL patients were initially diagnosed and followed at St-Sacrement and later at Enfant-Jésus hospitals of the CHU de Québec. Diagnostic criteria were (a) a persistent CD19^+^/CD5^−^ B cell lymphocytosis of at least 6 months' duration with a normal *κ*/*λ* ratio; (b) a polyclonal increase in the serum IgM concentration; (c) the presence on the blood smear of binucleated lymphocytes as previously described [11, 28]; and (d) the presence of multiple bcl-2/Ig gene rearrangements in peripheral blood lymphocytes. Blood was collected from all patients after informed consent was obtained. This study has been approved by the Research Ethics Board of the CHU de Québec. Peripheral blood mononuclear cells (PBMCs) were obtained from healthy individuals following routine platelet collection by recovering the cells from leukoreduction chambers, as described previously [32]. Consequently, comparison against samples obtained from healthy individuals participating in Héma-Québec's study was approved by Héma-Québec's Research Ethic Committee and all these samples were obtained following each individual's informed consent.

### 2.2. Isolation of Human Peripheral B Cells from PPBL Samples

PBMCs were isolated from peripheral blood by Ficoll-paque density gradient centrifugation (Amersham Pharmacia Biotech, Baie d'Urfé, Canada), suspended in freezing medium (Roswell Park Memorial Institute (RPMI) medium (Gibco-BRL, Burlington, Ont, Canada)), supplemented with 20% fetal bovine serum supplemented with 5% DMSO (FBS; Hyclone, Logan, UT, USA), and kept frozen in liquid nitrogen. B cells, from patients or from healthy controls, were purified by negative selection from thawed PBMCs cryopreserved for 3 months or less, using the StemSep CD19 mixture according to the manufacturer's instructions (Stem Cell Technologies, Vancouver, Canada) [30]. Purified human B cells were 95% or more CD19^+^ as determined by flow cytometry.

### 2.3. *In Vitro* Stimulation of Human B Cells with CD154^+^ Adherent Cells

L4.5 cells originate from a genetically modified L929 cell line (CCL-1, American Type Culture Collection, Manassas, VA) and express about 21,000 ± 4000 CD154 molecules per cell [30, 33]. Purified B cells from PPBL patients and controls (~2.5 × 10^5^ cells/mL) were seeded in Primaria plates (BD Biosciences, Mountain View, CA) in the presence of gamma-irradiated (75 Gy/7500 rad) L4.5 cells in a ratio of either 3 or 25 B cells per L4.5 cell, corresponding to high and low stimulations, respectively [30]. B cells were cultured in Iscove's modified Dulbecco's medium (IMDM) supplemented with 10% ultralow IgG FBS containing 10 *μ*g/mL insulin, 5.5 *μ*g/mL transferrin, 6.7 ng/mL sodium selenite (all from Invitrogen, Burlington, ON, Canada), and a mixture of cytokines, namely, 5 ng/mL IL-2 (~50 U/mL), 40 ng/mL IL-10 (~20 U/mL) (both from PeproTech, Rocky Hill, NJ, USA), and 3.5 ng/mL IL-4 (100 U/mL) (R&D Systems, Minneapolis, MN, USA). Three separate culture experiments were performed, each consisting of cells purified from 1 healthy control and 2 patients. Cell counts and viability were evaluated in triplicate by Trypan blue dye exclusion. Cultured B cells were always ≥95% CD19^+^ and, unless otherwise specified, viability was >90%.

### 2.4. Flow Cytometry Analysis

Allophycocyanin-conjugated anti-CD27 and anti-IgG, PerCP-cyanin 5.5-conjugated anti-CD19, and PE-conjugated anti-IgD and their conjugated isotype controls were all from BD Biosciences. Polyvalent goat IgG FITC-anti-IgM antibodies were from The Jackson Laboratory (Mississauga, Ontario, Canada). All stainings were performed using 1 *μ*g of each Ab for 1 × 10^6^ cells. Cells were fixed with 2% paraformaldehyde. Isotype-matched control Ab staining was >95% double-negative cells. Regions containing dead cells were delineated using 7-amino-actinomycin D staining, following manufacturer's instructions (BD Biosciences). Analyses were done by gating ≥10,000 cells with a FACSCalibur Flow cytometer and the CellQuest Pro software (BD Biosciences). Data were subsequently analyzed with FCS Express II software (De Novo Software, Thornhill, ON, Canada).

### 2.5. Ig-Secretion Rate

The IgG and IgM secretion rates were determined on either day 13 or 14. Briefly stated, cells were harvested, washed with PBS, and seeded at 1-2 × 10^6^ cells/mL in bare IMDM medium. Supernatants were collected after 18 to 22 hours and IgG and IgM concentrations were determined by standard enzyme-linked immunoadsorbent assay (ELISA), as previously described [29]. When indicated, IgG and IgM contents were also determined by ELISA in culture supernatants.

## 3. Results

### 3.1. Patient Profiles

The six patients studied were all female and displayed the clinical features associated with PPBL (Table 1). As described earlier [19], these were a higher than normal circulating lymphocyte count (*n* = 2, 4 to 6,4 × 10^9^/L), due to an increased number of B cells, and a polyclonal increase in their serum IgM (*n* = 3, 8 to 12,1 g/L). Their *κ*/*λ* ratio was consistent with a polyclonal origin of the lymphoproliferation in all cases (data not shown). A* Bcl2/Ig* gene rearrangement was present in everyone (Table 1). All patients were cigarette smokers, either presently or previously.

### 3.2. Phenotypic Characterization of Peripheral B Cells from Patients

**Figure 1:**
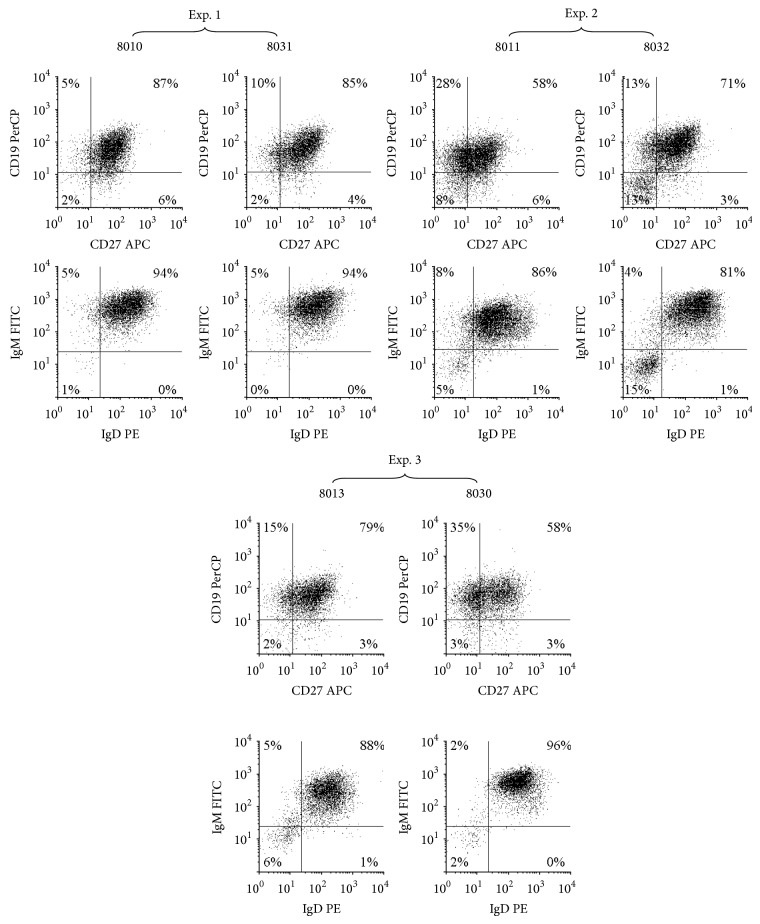
Phenotypes of B cells isolated from the blood samples of six patients with persistent polyclonal B cell lymphocytosis. Flow cytometry analysis of CD19, CD27, IgD, and IgM expression was done on purified peripheral B cells. More than 95% of purified cells were CD19^+^CD40+ cells for all these patient samples. All analyses were done as described in methods.

Flow cytometry was done on purified B cells from each of the six patients to evaluate CD19, CD27, IgD, and IgM expression (Figure 1). As previously reported [11], 73% ± 13% (mean ± SD; range of 58% to 87%) of the CD19^+^ B cell populations were characterized by CD27 positivity and 90% ± 6% (mean ± SD; range of 81% to 96%) expressed IgD and IgM. All B cells from PPBL patients also expressed CD40 (data not shown). Such high frequency of CD27^+^IgM^+^IgD^+^ B cell subset in the purified CD19^+^ cells was as expected and in agreement with the clinical data of our PPBL patients.

### 3.3. PPBL B Cells Respond to Low CD40 Stimulation

**Figure 2:**
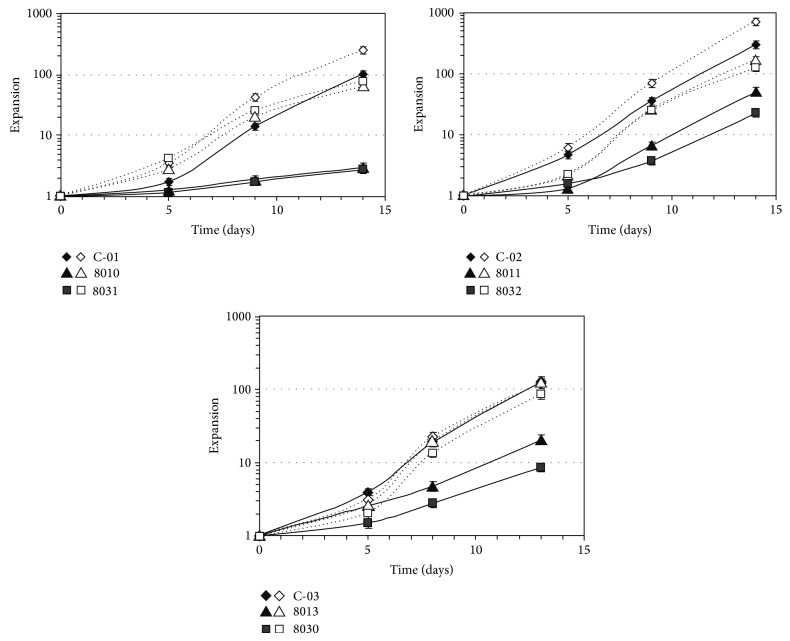
PPBL B cells proliferate following low CD154 interaction. Purified B cells isolated from samples obtained from healthy individuals (C-01, C-02, and C-03) and from patients with PPBL (8010, 8011, 8013, 8030, 8031, and 8032) were activated* in vitro* using a low level (open symbols; dashed line) or a high level (filled symbols; plain line) of CD154 interaction in the presence of cytokines, as indicated in methods. Expansion factors were evaluated using viable cell counts performed in triplicate at the indicated times. Error bars can be smaller than symbols.

B cells from PPBL patients and healthy individuals were submitted to culture with high (3 B cells per L4.5 cell) and low (25 B cells per L4.5 cell) stimulation conditions and their proliferation was monitored for 13 to 14 days (Figure 2). As previously observed, the response of PPBL B cells to a high CD154 interaction was still 6- to 30-fold lower than that of control B cells, presumably due to their high frequency of CD27^+^IgM^+^IgD^+^ cells. Conversely, all PPBL B cell samples responded to the low stimulation conditions, with an expansion factor of anywhere between 3 and 20 times higher than that observed in the high CD40-CD154 interaction experiments. However, the response of PPBL B cells to a low CD40 interaction was lower than that of control B cells, which contained a normal ratio of naive CD27^−^ and memory CD27^+^ B cells (data not shown). It is likely that the degree of proliferation of the dominant CD27^+^IgM^+^IgD^+^ B cell subpopulation observed in our patients is much lower than the otherwise more prominent CD27^−^ B cell subpopulations of our controls [11].

### 3.4. Evolution of PPBL B Cells following Long-Term CD40 Stimulation

**Figure 3:**
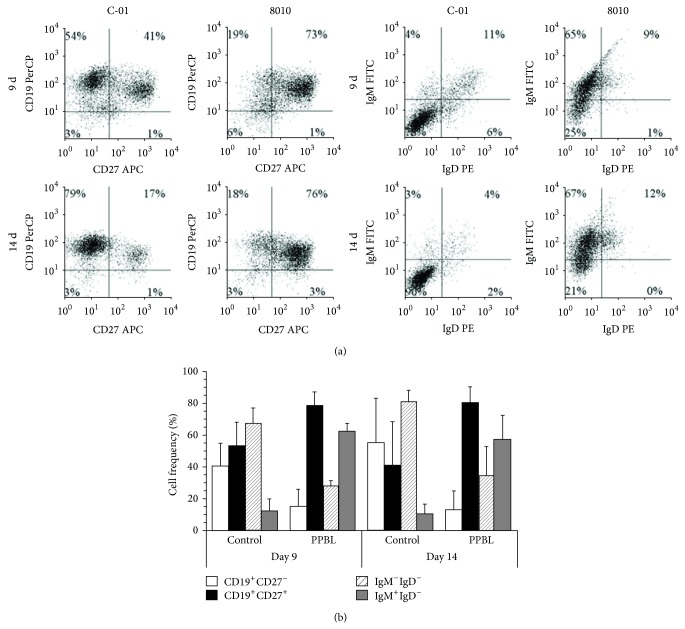
Phenotypes of PPBL B cells following low CD154 interaction. CD19, CD27, IgD, and IgM expressions were evaluated by flow cytometry on B cells receiving a low level of CD154 interaction (shown in Figure 2), at days 8 to 9 (9 d) or 13 to 14 (14 d), as indicated. (a) Phenotypic profiles are shown for one control (C-01) and one PPBL sample (8010). (b) The frequencies of cells in the three control samples and the 6 PPBL samples are shown for the main subsets of CD19^+^CD27^−^, CD19^+^CD27^+^, IgM^−^IgD^−^, and IgM^+^IgD^−^ cells, as illustrated in. (a) Data is presented as mean ± SD.

The phenotypes of PPBL B cells following low CD40 stimulation were monitored for CD19, CD27, IgM, and IgD expression on days 9 and 14. Phenotypic profiles for one representative PPBL sample and an example of control B cells are presented below (Figure 3(a)). The frequencies for CD19^+^CD27^−^, CD19^+^CD27^+^, IgM^−^IgD^−^, and IgM^+^IgD^−^ cells are shown as the mean values for all PPBL samples (*n* = 6) and the three controls (Figure 3(b)). Phenotypic evolution of the CD19^+^ B cells, similar in all PPBL samples, differed from that of control B cells by showing a higher frequency of CD27^+^ and IgM^+^IgD^−^ cells (Figures 3(a) and 3(b)). In all samples, two main phenotypes, corresponding to CD19^+^CD27^−^ and CD19^+^CD27^+^ cells, were observed during days 9 through 14. Based upon CD27 expression, the evolution of B cells from PPBL patients, following low CD40 stimulation, appeared similar to that of B cells from healthy controls. At rest, 81 to 96% of PPBL B cells were IgD^lo^IgM^+^ (Figure 1), a phenotype similar to that of normal marginal zone B cells [11, 34]. Following CD40 stimulation, surface IgD almost vanished in all PPBL B cell samples (Figures 3(a) and 3(b)). Additionally, the frequency of total IgM^+^ B cells decreased in controls, from 88 to 95% (day 0) to less than 10% of CD19^+^ cells (day 14), while remaining at 57% ± 15% in PPBL samples. Overall, these results indicate that the response of PPBL B cells to CD40 stimulation leads to phenotype changes that are quite similar to those observed in normal B cell populations but that remains biased towards growth of IgM^+^ B cell subsets.

### 3.5. PPBL B Cells Are Able to Switch to and to Secrete IgG

**Figure 4:**
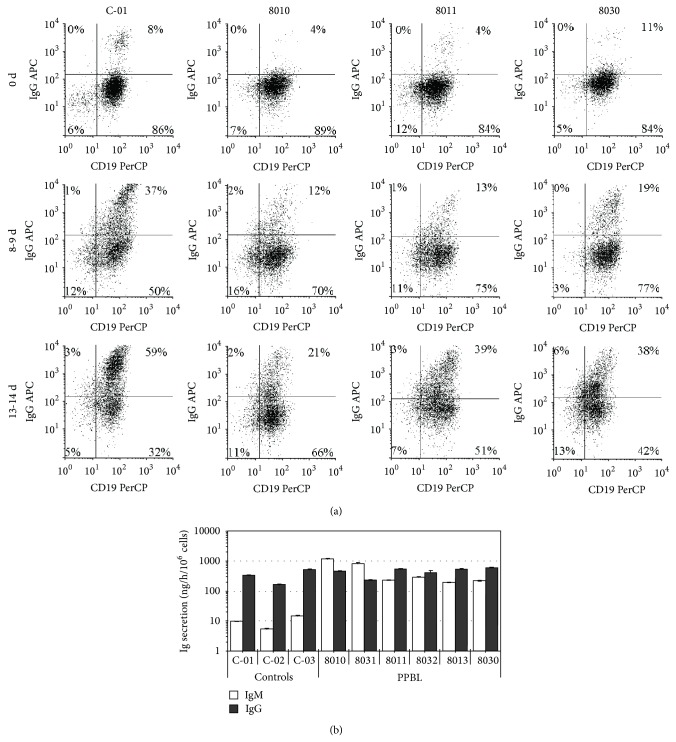
PPBL B cells can morph into IgG secreting cells following long-term CD154 stimulation. B cells stimulated with a low level of CD154 interaction (Figure 2) were analyzed for their ability to express and secrete IgG. (a) The proportion of IgG^+^ within CD19^+^ cells before (0 d) and after exposure to a low level of CD154 interaction (8 d and 13 d for 8030 and 9 d and 14 d for 8010 and 8011) was determined by flow cytometry. These phenotypes are representative of the six analyzed samples. (b) IgG and IgM secretion rates were determined at the end of the culture periods on all samples (patients and healthy controls).

**Figure 5:**
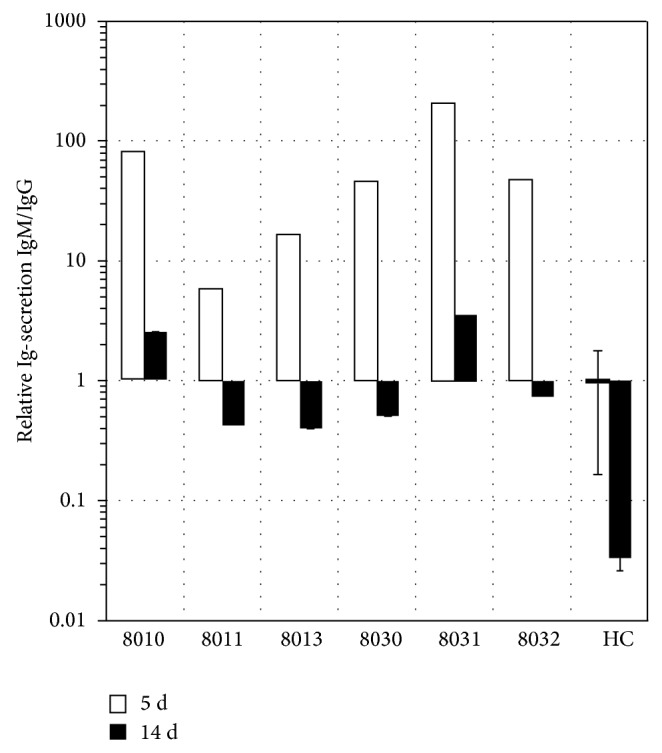
Isotype switching during culture of PPBL B cells with low CD154 interaction. The IgG and IgM secretion ratios of PPBL CD19^+^ cells stimulated with a low level of CD154 interaction were determined by ELISA in supernatant collected after 5 days (5 d) and evaluated on day 14 (14 d), for all patient samples. Four healthy controls submitted to identical culture conditions (HC) were analyzed similarly and data is presented as the mean ± SD.

Based on the above results, flow cytometry analyses of CD40-activated PPBL B cells were performed in order to monitor their expression of IgG (Figure 4(a)). It was thus found that a heightened proportion of IgM^−^IgD^−^ B cells correlated quite precisely with the emergence of IgG^+^ B cells. Furthermore, high IgG secretion rates, similar to those observed in control B cells, were observed in CD40-activated B cells from the six PPBL patient samples (Figure 4(b)). Conversely, IgM secretion rates within PPBL B cells were higher than those seen in control B cells, in concordance with the higher proportion of IgM^+^ cells. In addition, the capacity of PPBL B cells to switch isotypes and to secrete IgG suggests that their* in vitro* response to CD40 is similar to that of normal B cells. Another proof of the* in vitro* isotype switching capacity of PPBL B cells is in the observed difference between IgM/IgG ratios at days 5 and 14 for cultures in a low interaction CD40-CD154 medium. On day 5, the IgM/IgG ratio of PPBL B cells varied between 6 and 205, whereas at the end of the cultures (day 14), the IgM/IgG ratio was nearly or completely reversed in favour of IgG (0,4 to 3,5) (Figure 5). These observations are in agreement with the reported isotype switching capacity of normal CD27^+^IgD^+^IgM^+^ human B cells [35].

## 4. Discussion

The importance of a functional CD40 molecule in B cell development, proliferation, and immunoglobulin production is well illustrated by the X-linked hyper-IgM syndrome, which is the outcome of an inadequate interaction between CD40 on B lymphocytes and its ligand, CD154, presented by activated T cells [36, 37]. This deficiency affects the interaction between activated CD4^+^ T cells and all other cell types expressing CD40, namely, B cells, dendritic cells, monocytes/macrophages, platelets, and activated endothelial and epithelial cells. This inherited condition is characterized by a defective class-switch recombination, resulting in normal or increased levels of serum IgM associated with deficiencies of IgG, IgA, and IgE. Moreover, the lymphoid organs of affected individuals are devoid of germinal centres and they are unable to develop memory B cells in response to T-dependent antigens [38]. The characteristics associated with this disorder are partially reminiscent of those observed in patients affected with persistent polyclonal B cell lymphoproliferation. Indeed, patients with PPBL showed elevated serum IgM and polyclonal B cell proliferation. Consequently, we asked ourselves whether or not there was a similar pathophysiology between these two disorders, specifically, a defect in the CD40-CD154 signaling pathway. Although many cases of hyper-IgM syndrome are related to genetic abnormalities affecting CD154 and preventing its interaction with its receptor [39–41], a subset of patients has been described in whom the disorder rather stems from the presence of defects in the CD40-induced B cell activation pathway [42, 43]. When B lymphocytes isolated from this subset of hyper-IgM syndrome patients are stimulated* in vitro* in the CD40-dependent cell culture system in the presence of an intact ligand, they are still unable to proliferate and undergo immunoglobulin isotype switching. Likewise, we had already demonstrated that B lymphocytes isolated from PPBL patients did not respond to the expansion signal delivered through CD40 when the level of interaction between CD40 and CD154 was high, indicating a possible defect in the CD40 signaling pathway, despite normal expression, sequencing, and tyrosine phosphorylation of CD40 [28]. It was later demonstrated that PPBL B cells were in essence memory B cells harbouring the CD27^+^IgM^+^IgD^+^ immunotype [11, 21]. The peripheral CD19^+^CD27^+^ memory B cells from normal individuals were then reported to be unresponsive to high-level CD40 stimulation [29]. However, it has been shown recently that a reduced intensity of CD40-CD154 interaction in the presence of IL-2, IL-4, and IL-10 results in the proliferation, expansion, and immunoglobulin secretion of normal memory CD19^+^CD27^+^IgM^+^ B cells [30]. We thus performed a culture of PPBL B cells in the low-interaction CD40-CD154 medium and obtained a proliferation of the CD19^+^CD27^+^IgM^+^ B cells that was 6 to 20 times higher than that observed with the high CD154 interaction medium. We demonstrated that these lymphocytes were capable of proliferating* in vitro* in the presence of precise and optimal culture conditions, that is, a low level of interaction between CD40 and CD154 (3 cells expressing the CD154 ligand for 25 B lymphocytes) in the presence of IL-2, 4, and 10. We can now confirm that PPBL patients did not demonstrate any defect in the CD40-induced signaling pathway.

The following interesting observation was made: the CD19^+^IgG^+^ cell population, encompassing globally less than 5% of the cell population at the beginning of the culture in PPBL patients, increased beyond 25% on day 14. Meanwhile, we observed the emergence of a CD19^+^CD27^−^ cell population and the disappearance of surface IgD as in normal controls. Such downregulation of IgD is traditionally observed in B cell subsets following their activation within germinal center [44] and is usually associated with further secretion of immunoglobulin. Similarly, when cultured under proper condition, B cells from patients with PPBL showed a heightened proportion of IgM^−^IgD^−^ B cells along with an emergence of IgG^+^ B cells and high immunoglobulin secretion. By dosing immunoglobulin secretion from cultured controls and patients' B cells, we observed a much larger immunoglobulin secretion rate in the low interaction culture system than in the high interaction CD40-CD154 medium. Those results were expected in controls, since we already knew that a low level interaction medium promotes differentiation and secretion of memory B cells whereas a high interaction medium preferentially stimulates proliferation of normal B cells [30]. In the low-level CD40-CD154 interaction medium, we observed much larger IgG and IgM secretion rate from PPBL B cells than from healthy control B cells. We believe that this is attributable to the IgM-rich B lymphocyte subpopulations seen in large quantities, relative to controls, in PPBL patient. It is also possible that PPBL B cells were able to differentiate faster than those from controls.

The high proportion of IgM^+^IgD^+^ B cell population and increased IgM levels in PPBL patients suggest a difficulty in normally completing isotype switching. On day 14, immunoglobulin isotype analysis showed higher IgG than IgM levels. These results strongly demonstrate the capacity of* in vitro* isotype switching of PPBL B cells. We also observed that IgG preferential secretion occurred in the low-level CD40-CD154 medium, thus reinforcing the* in vitro* isotype switching hypothesis.

## 5. Conclusion

In summary, we have shown throughout this study that PPBL B cells could proliferate in a CD40-CD154 culture system under proper conditions and that proliferation also results in IgM and IgG secretion, all of which indicating an adequate CD40 signaling pathway. Moreover, this report provides the first evidence of* in vitro* immunoglobulin isotype switching of CD19^+^CD27^+^IgM^+^ B cells from PPBL, while denoting that this capacity may be impaired* in vivo* [45]. These results also indicate that these same CD19^+^CD27^+^IgM^+^ B cells are not as abnormal as we initially believed and thus that PPBL may arise, as recently proposed, from a deregulation of the microenvironment [46] or from a defect in a different B cell activation pathway, resulting in extensive proliferation.

## Figures and Tables

**Table 1:** Clinical data of PPBL patients.

Characteristics	Patients					
	8010	8011	8013	8030	8031	8032
Age (years)	50	47	59	69	56	49

Lymphocytosis (×10^9^/L)	6,4	2,5	3,9	2,8	4,4	2,4

Lymphocytosis (% total leukocytosis)	58,4	32,1	36,5	42,5	56,6	48,7

B cells/*μ*L	5250	1550	910	1200	2240	760

Serum IgM (g/L)	12,1	7,8	3,8	9,6	9,5	7,3

Number of *BCL-2/Ig* rearrangements^a^	3	5	3	7	5	5

HLA	A 2, 9 B 14, 44 Dr 2, 5	A 9, 30 B 13, 14 Dr 5, 13	A 9, 39 B 14, 35 Dr 5, 13	A 1, 3 B 7, 57 Dr 7, 14	ND	A 11, 29 B 35, 44 Dr 7, 14

Cigarette smoking^b^	Yes	Stopped × 7 years	Yes	Stopped × 3 years	Yes	Stopped × 5 years

^a^ *BCL-2/Ig* gene rearrangement was determined as previously described by Delage et al., 2001 [19].

^b^Information was reported on day of blood collection.

## References

[1] Gordon D. S., Jones B. M., Browning S. W., Spira T. J., Lawrence D. N. (1982). Persistent polyclonal lymphocytosis of B lymphocytes. *The New England Journal of Medicine*.

[2] Mossafa H., Malaure H., Maynadie M., Valensi F., Schillinger F., Garand R., Jung G., Flandrin G., Troussard X. (1999). Persistent polyclonal B lymphocytosis with binucleated lymphocytes: a study of 25 cases. Groupe Francais d'Hematologie Cellulaire. *The British Journal of Haematology*.

[3] Callet-Bauchu E., Renard N., Gazzo S., Poncet C., Morel D., Pagès J., Salles G., Coeur P., Felman P. (1997). Distribution of the cytogenetic abnormality +i(3)(q10) in persistent polyclonal B-cell lymphocytosis: a FICTION study in three cases. *British Journal of Haematology*.

[4] Cornet E., Lesesve J. F., Mossafa H., Sébahoun G., Levy V., Davi F., Troussard X. (2009). Long-term follow-up of 111 patients with persistent polyclonal B-cell lymphocytosis with binucleated lymphocytes. *Leukemia*.

[5] Troussard X., Mossafa H., Flandrin G., Machii T., Yamaguchi M., Inoue R., Tokumine Y., Kuratsune H., Nagai H., Fukuda S., Furuyama K., Yamada O., Yawata Y., Kitani T. (1997). Identity between hairy B-cell lymphoproliferative disorder and persistent polyclonal B lymphocytosis?. *Blood*.

[6] Feugier P., De March A. K., Lesesve J. F., Monhoven N., Dorvaux V., Braun F., Grégoire M. J., Jonveaux P., Lederlin P., Béné M. C., Labouyrie E. (2004). Intravascular bone marrow accumulation in persistent polyclonal lymphocytosis: a misleading feature for B-cell neoplasm. *Modern Pathology*.

[7] Carstairs K. C., Francombe W. H., Scott J. G., Gelfand E. W. (1985). Persistent polyclonal lymphocytosis of B lymphocytes, induced by cigarette smoking?. *The Lancet*.

[8] Casassus P., Lortholary P., Komarover H., Lejeune F., Hors J. (1987). Cigarette smoking-related persistent polyclonal B lymphocytosis. A premalignant state. *Archives of Pathology and Laboratory Medicine*.

[9] Chan M. A., Benedict S. H., Carstairs K. C., Francombe W. H., Gelfand E. W. (1990). Expansion of B lymphocytes with an unusual immunoglobulin rearrangement associated with atypical lymphocytosis and cigarette smoking. *The American Journal of Respiratory Cell and Molecular Biology*.

[10] Delannoy A., Djian D., Wallef G., Deneys V., Fally P., Martiat P., Michaux J. L. (1993). Cigarette smoking and chronic polyclonal B-cell lymphocytosis. *Nouvelle Revue Francaise d'Hematologie*.

[11] Loembe M. M., Néron S., Delage R., Darveau A. (2002). Analysis of expressed ${\text{V}}_{\text{H}}$ genes in persistent polyclonal B cell lymphocytosis reveals absence of selection in CD27^+^IgM^+^IgD^+^ memory B cells. *European Journal of Immunology*.

[12] Küppers R., Klein U., Hansmann M.-L., Rajewsky K. (1999). Cellular origin of human B-cell lymphomas. *The New England Journal of Medicine*.

[13] Troussard X., Mossafa H., Valensi F., Maynadie M., Schillinger F., Bulliard G., Malaure H., Flandrin G. (1997). Polyclonal lymphocytosis with binucleated peripheral lymphocytes. Morphological, immunological, cytogenetic and molecular analysis in 15 cases. *Presse Medicale*.

[14] Agrawal S., Matutes E., Voke J., Dyer M. J. S., Khokhar T., Catovsky D. (1994). Persistent polyclonal B-cell lymphocytosis. *Leukemia Research*.

[15] Roy J., Ryckman C., Bernier V., Whittom R., Delage R. (1998). Large cell lymphoma complicating persistent polyclonal B cell lymphocytosis. *Leukemia*.

[16] Lawlor E., Murray M., O'Briain D. S., Blaney C., Foroni L., Sarsfield P., Condell D., Sullivan F., McCann S. R. (1991). Persistent polyclonal B lymphocytosis with Epstein-Barr virus antibodies and subsequent malignant pulmonary blastoma. *Journal of Clinical Pathology*.

[17] Schmidt-Hieber M., Burmeister T., Weimann A., Nagorsen D., Hofmann W. K., Thiel E., Schwartz S. (2008). Combined automated cell and flow cytometric analysis enables recognition of persistent polyclonal B-cell lymphocytosis (PPBL), a study of 25 patients. *Annals of Hematology*.

[18] Troussard X., Cornet E., Lesesve J. F., Kourel C., Mossafa H. (2008). Polyclonal B-cell lymphocytosis with binucleated lymphocytes (PPBL). *OncoTargets and Therapy*.

[19] Delage R., Jacques L., Massinga-Loembe M., Poulin J., Bilodeau D., Mignault C., Leblond P. F., Darveau A. (2001). Persistent polyclonal B-cell lymphocytosis: further evidence for a genetic disorder associated with B-cell abnormalities. *British Journal of Haematology*.

[20] Delage R., Roy J., Jacques L., Bernier V., Delàge J.-M., Darveau A. (1997). Multiple bcl-2/Ig gene rearrangements in persistent polyclonal B-cell lymphocytosis. *British Journal of Haematology*.

[21] Himmelmann A., Gautschi O., Nawrath M., Bolliger U., Fehr J., Stahel R. A. (2001). Persistent polyclonal B-cell lymphocytosis is an expansion of functional IgD^+^CD27^+^ memory B cells. *British Journal of Haematology*.

[22] Salcedo I., Campos-Caro A., Sampalo A., Reales E., Brieva J. A. (2002). Persistent polyclonal B lymphocytosis: an expansion of cells showing IgVH gene mutations and phenotypic features of normal lymphocytes from the CD27+ marginal zone B-cell compartment. *British Journal of Haematology*.

[23] Del Giudice I., Pileri S. A., Rossi M., Sabattini E., Campidelli C., Starza I. D., De Propris M. S., Mancini F., Perrone M. P., Gesuiti P., Armiento D., Quattrocchi L., Tafuri A., Amendola A., Mauro F. R., Guarini A., Foà R. (2009). Histopathological and molecular features of persistent polyclonal B-cell lymphocytosis (PPBL) with progressive splenomegaly. *British Journal of Haematology*.

[24] Weill J.-C., Weller S., Reynaud C.-A. (2009). Human marginal zone B cells. *Annual Review of Immunology*.

[25] Tangye S. G., Good K. L. (2007). Human IgM^+^CD27^+^ B cells: memory B cells or “memory” B cells?. *The Journal of Immunology*.

[26] Van Kooten G., Banchereau J. (2000). CD40-CD40 ligand. *Journal of Leukocyte Biology*.

[27] Banchereau J., de Paoli P., Vallé A., Garcia E., Rousset F. (1991). Long-term human B cell lines dependent on interleukin-4 and antibody to CD40. *Science*.

[28] Loembé M. M., Lamoureux J., Deslauriers N., Darveau A., Delage R. (2001). Lack of CD40-dependent B-cell proliferation in B lymphocytes isolated from patients with persistent polyclonal B-cell lymphocytosis. *British Journal of Haematology*.

[29] Fecteau J. F., Néron S. (2003). CD40 stimulation of human peripheral B lymphocytes: distinct response from naïve and memory cells. *Journal of Immunology*.

[30] Néron S., Racine C., Roy A., Guérin M. (2005). Differential responses of human B-lymphocyte subpopulations to graded levels of CD40-CD154 interaction. *Immunology*.

[31] Fecteau J. F., Roy A., Néron S. (2009). Peripheral blood CD27^+^ IgG^+^ B cells rapidly proliferate and differentiate into immunoglobulin-secreting cells after exposure to low CD154 interaction. *Immunology*.

[32] Néron S., Thibault L., Dussault N. (2007). Characterization of mononuclear cells remaining in the leukoreduction system chambers of apheresis instruments after routine platelet collection: a new source of viable human blood cells. *Transfusion*.

[33] Néron S., Pelletier A., Chevrier M.-C., Monier G., Lemieux R., Darveau A. (1996). Induction of LFA-1 independent human B cell proliferation and differentiation by binding of CD40 with its ligand. *Immunological Investigations*.

[34] Weller S., Braun M. C., Tan B. K., Rosenwald A., Cordier C., Conley M. E., Plebani A., Kumararatne D. S., Bonnet D., Tournilhac O., Tchernia G., Steiniger B., Staudt L. M., Casanova J.-L., Reynaud C.-A., Weill J.-C. (2004). Human blood IgM “memory” B cells are circulating splenic marginal zone B cells harboring a prediversified immunoglobulin repertoire. *Blood*.

[35] Werner-Favre C., Bovia F., Schneider P. (2001). IgG subclass switch capacity is low in switched and in IgM-only, but high in IgD^+^IgM^+^, post-germinal center (CD27^+^) human B cells. *European Journal Immunology*.

[36] Lougaris V., Badolato R., Ferrari S., Plebani A. (2005). Hyper immunoglobulin M syndrome due to CD40 deficiency: clinical, molecular, and immunological features. *Immunological Reviews*.

[37] Longo N. S., Lugar P. L., Yavuz S., Zhang W., Krijger P. H. L., Russ D. E., Jima D. D., Dave S. S., Grammer A. C., Lipsky P. E. (2009). Analysis of somatic hypermutation in X-linked hyper-IgM syndrome shows specific deficiencies in mutational targeting. *Blood*.

[38] Vogel L. A., Noelle R. J. (1998). CD40 and its crucial role as a member of the TNFR family. *Seminars in Immunology*.

[39] Allen R. C., Armitage R. J., Conley M. E., Rosenblatt H., Jenkins N. A., Copeland N. G., Bedell M. A., Edelhoff S., Disteche C. M., Simoneaux D. K., Fanslow W. C., Belmont J., Spriggs M. K. (1993). CD40 ligand gene defects responsible for X-linked hyper-IgM syndrome. *Science*.

[40] DiSanto J. P., Bonnefoy J. Y., Gauchat J. F., Fischer A., de Saint Basile G. (1993). CD40 ligand mutations in x-linked immunodeficiency with hyper-IgM. *Nature*.

[41] Korthauer U., Graf D., Mages H. W., Briere F., Padayachee M., Malcolm S., Ugazio A. G., Notarangelo L. D., Levinsky R. J., Kroczek R. A. (1993). Defective expression of T-cell CD40 ligand causes X-linked immunodeficiency with hyper-IgM. *Nature*.

[42] Conley M. E., Larché M., Bonagura V. R., Lawton A. R., Buckley R. H., Fu S. M., Coustan-Smith E., Herrod H. G., Campana D. (1994). Hyper IgM syndrome associated with defective CD40-mediated B cell activation. *Journal of Clinical Investigation*.

[43] Durandy A., Hivroz C., Mazerolles F., Schiff C., Bernard F., Jouanguy E., Revy P., DiSanto J. P., Gauchat J. F., Bonnefoy J. Y., Casanova J. L., Fischer A. (1997). Abnormal CD40-mediated activation pathway in B lymphocytes from patients with hyper-IgM syndrome and normal CD40 ligand expression. *The Journal of Immunology*.

[44] Pascual V., Liu Y.-J., Magalski A., de Bouteiller O., Banchereau J., Capra J. D. (1994). Analysis of somatic mutation in five B cell subsets of human tonsil. *The Journal of Experimental Medicine*.

[45] Hafraoui K., Moutschen M., Smet J., Mascart F., Schaaf-Lafontaine N., Fillet G. (2009). Selective defect of anti-pneumococcal IgG in a patient with persistent polyclonal B cell lymphocytosis. *European Journal of Internal Medicine*.

[46] Berkowska M. A., Grosserichter-Wagener C., Adriaansen H. J., de Ridder D., Mirani-Oostdijk K. P., Agteresch H. J., Böttcher S., Orfao A., van Dongen J. J. M., van Zelm M. C. (2014). Persistent polyclonal B-cell lymphocytosis: extensively proliferated CD27+IgM+IgD+ memory B cells with a distinctive immunophenotype. *Leukemia*.

